# Factors influencing outcomes in selective neck dissection in 661 patients with head and neck squamous cell carcinoma

**DOI:** 10.1186/s12893-022-01644-6

**Published:** 2022-05-19

**Authors:** Mattis Bertlich, Nina Zeller, Saskia Freytag, Jennifer L. Spiegel, Bernhard G. Weiss, Martin Canis, Frank Haubner, Friedrich Ihler

**Affiliations:** 1 Department of Dermatology and Allergy, University Hospital, Ludwig-Maximilians-University of Munich, Marchioninistr. 15, 81377 Munich, Germany; 2 Department of Otorhinolaryngology, Klinikum Frankfurt Höchst, Gotenstraße 6-8, 65929 Frankfurt am Main, Germany; 3 Epigenetics and Genomics, Harry Perkins Institute of Medical Research, Nedlands, WA Australia; 4 Department of Otorhinolaryngology, University Hospital, Ludwig-Maximilians-University of Munich, Marchioninistr. 15, 81377 Munich, Germany; 5 Department of Otorhinolaryngology, Head and Neck Surgery, Greifswald University Medicine, University of Greifswald, Fleischmannstraße 8, 17475 Greifswald, Germany

**Keywords:** Head and neck squamous cell carcinoma, Neck dissection, Selective neck dissection

## Abstract

**Background:**

Selective neck dissection (SND) is the surgical treatment of choice in squamous cell carcinoma of the head and neck (HNSCC) with suspected or manifest metastases in the cervical lymph nodes. For SND to be successful, treated lymph node levels should be selected according to anatomic considerations and the extent of the disease. Aim of this study was to identify neck dissection levels that had an impact on individual prognosis.

**Methods:**

We conducted a retrospective review of SND as part of primary treatment of HNSCC. Overall survival (OS) and regional control rates (RCR) were calculated for all patients treated at one academic tertiary referral center.

**Results:**

661 patients with HNSCC were included, 644 underwent ipsilateral and 319 contralateral SND. Average follow-up was 78.9 ± 106.4 months. 67 (10.1%) patients eventually developed nodal recurrence. Tumor sites were oral cavity (135), oropharynx (179), hypopharynx (118) and larynx (229). Tumor categories pT1–pT4a, and all clinical and pathological nodal categories were included. Multivariate analysis indicated improved OS rates for patients undergoing SND in ipsilateral levels I and V as well as level III contralaterally. Analysis for tumor origin showed that SND in ipsilateral level I showed significantly improved OS in HNSCC of the oral cavity.

**Conclusion:**

The dissection of ipsilateral level I in oral cavity cancer was of particular relevance in our exploratory, retrospective analysis. To clarify the relevance for the determination of the extent of SND, this should be investigated prospectively in a more homogenous patient cohort.

## Introduction

Clinical and pathological presence of lymph node metastasis is—apart from distant metastasis—the most important prognostic factor in patients suffering from squamous cell carcinoma of the head and neck (HNSCC) [1]. It has been reported that only one affected lymph node may decrease overall survival rates by as much as 50% [2]. Consequently, adequate treatment of the lymphatic regions is paramount to obtain an adequate oncologic result.

Generally speaking, the neck is commonly treated in the same manner as the primary tumor—i.e., if the tumor was treated surgically, so is the neck, with or without postoperative radio-(chemo-)therapy. In turn, if a tumor is treated by definitive chemoradiation, the neck is commonly radiated as well. If the neck is treated surgically, selective neck dissection (SND) is the treatment of choice, [3] except in cases where non-lymphatic structures are involved by tumor spread and have to be removed by radical or modified radical neck dissection. Neck Dissection is the surgical treatment of choice in lymphatic affection in HNSCC and should be performed even in early and clinically inappareant stages [4].

While RND has been the treatment of choice for the past, focus in neck dissection has shifted to less invasive techniques in the past decades [5, 6]. In selective neck dissection (SND), many structures that are usually removed are preserved, including muscles, nerves and vascular structures. Moreover, one or several lymph node levels of the ipsi- or contralateral side are regularly spared. The assumption behind this approach is that the metastatic behavior of the primary tumor follows a predictable pattern, depending on where the tumor originated, and the treated regions are selected accordingly. The concept of predictable sequential regional spread in HNSCC has recently been confirmed for oral cavity squamous cell carcinoma by a comprehensive meta-analysis [7]. This makes neck dissection significantly less invasive and causes less short- and long-term side effects.

SND is to this day the surgical treatment of choice in patients with both likely nodal spread [8] as well as patients with clinically positive neck status [9]. However, decision making is still difficult when it comes to selective neck dissection. Hence, we conducted a retrospective analysis of factors influencing the outcomes of neck dissection in a large collective at a tertiary referral center.

## Materials and methods

### Ethics

The study at hand was conducted in accordance with the Declaration of Helsinki in its current form (seventh revision, 2013). The study was approved by the ethics committee at the University Medical Center of the Georg-August University Göttingen (Niedersachsen, Federal Republic of Germany) on January 10^th^ 2017 under the File No. DOK_200_2016. All study participants consented in written form to the analysis of medical data before treatment at the study center.

### Data acquisition

For data acquisition, the tumor database of the Department of Otorhinolaryngology of the University of Göttingen, established in 1986 by Wolfgang Steiner and coworkers, was used. This is a prospective database for all patients that had been primarily treated for HNSCC. Documented values included age, gender, tumor location, size, clinical TNM category, pathological TNM status, whether a neck dissection was performed, which levels were operated and the subsequent follow-up. Additionally, data was extracted from individual patient files. TNM-Categories were given according to the 6th edition from 2002.

### Inclusion and exclusion criteria

Patients with primary diagnosis of head and neck squamous cell carcinoma and surgical therapy in curative intention were considered for this analysis. Included were only patients with neck dissection as part of the initial therapy sequence. Exclusion criteria were cT4b category, distant metastasis at initial presentation, patients that did not undergo surgical therapy of the tumor and patients that suffered from a synchronous secondary malignant disease. Also, patients that developed a local recurrence during follow-up were excluded from the analysis.

### Treatment

All patients that were included in this study underwent transoral laser microsurgery (TLM) as primary treatment as established by Steiner and coworkers [10–12] with concomitant neck dissection. The dominance of TLM is particular for the local philosophy for management of HNSCC. In general, patients underwent SND of the ipsilateral levels II and III. If the primary tumor site was in the oral cavity, ipsilateral level I was included as well. In supraglottic laryngeal and hypopharyngeal tumor sites, levels II and III were treated on both sides. If tumor size was cT3 or cT4a of the oropharynx, hypopharynx or larynx, SND was performed in levels II, III and IV, respectively. If a cT3 or cT4a tumor was located in the oral cavity, levels I-IV were treated bilaterally. If any level showed suspicious lymph nodes, that region was included in the SND as well. Patients that underwent SND in other levels than stated previously were still included in the analysis. Treatment decisions including the addition of radiation and chemotherapy were made interdisciplinary. While surgical treatment took place at the study center only, radio- or chemoradiotherapy was administered at the facility nearest to the patient’s residence as it is common in the national health insurance system. The indication for adjuvant treatment was considerably strict, in particular during the early years of the long observation period. This most likely lead to an overrepresentation of more advanced cases in the group with adjuvant treatment in comparison to the international literature.

### Follow-up

Patients were scheduled for follow-up visits at the study center beginning 6 weeks after completion of initial therapy and quarterly thereafter. Follow-up-appointments were scheduled indefinitely if patients did not succumb to the disease. Although there was no recall if patients did not keep sequential appointments, this resulted in a considerable share of cases with a very long record after initial diagnosis.

### Calculation of overall survival and regional control rates

Outcome measures were chosen that reflected a successful course after initial tumor therapy. When calculating overall survival (OS), only death from any cause was counted as an event. This resulted in the percentage of surviving patients from all patients under investigation at any given timepoint. When calculating regional control rates (RCR), tumor associated death or lymph node recurrence were counted as events while patients that died from any cause apart from tumor associated death were considered as censored. Thereby, the share of living patients without tumor recurrence over time resulted.

### Statistics

Statistics were carried out using Project R for Mac (Build 3.4.1 for El Capitan, The R Project for Statistical Computing, http://www.r-project.org/) To estimate 5 and 10 year OS and RCR, Kaplan–Meier-estimates were used. To assess the influence of individual parameters on OS or RCR, COX-Regressions were used. To detect interactions of the effects between tumor site and side, additional COX-Regressions were performed. If p < 0.05, the results were considered to be statistically significant.

## Results

Overall, 1608 patients were in the database. Out of these, 661 patients met inclusion criteria. Average age at time of diagnosis was 56.8 ± 10.1 years, with 558 (84.4%) being male and 103 (15.6%) being female. Mean follow-up time was 78.9 ± 106.4 months (median 63.5, range 0.3–263.3 months; 25th and 75th percentile 33.7 and 112.0 months).

**Fig. 1:**
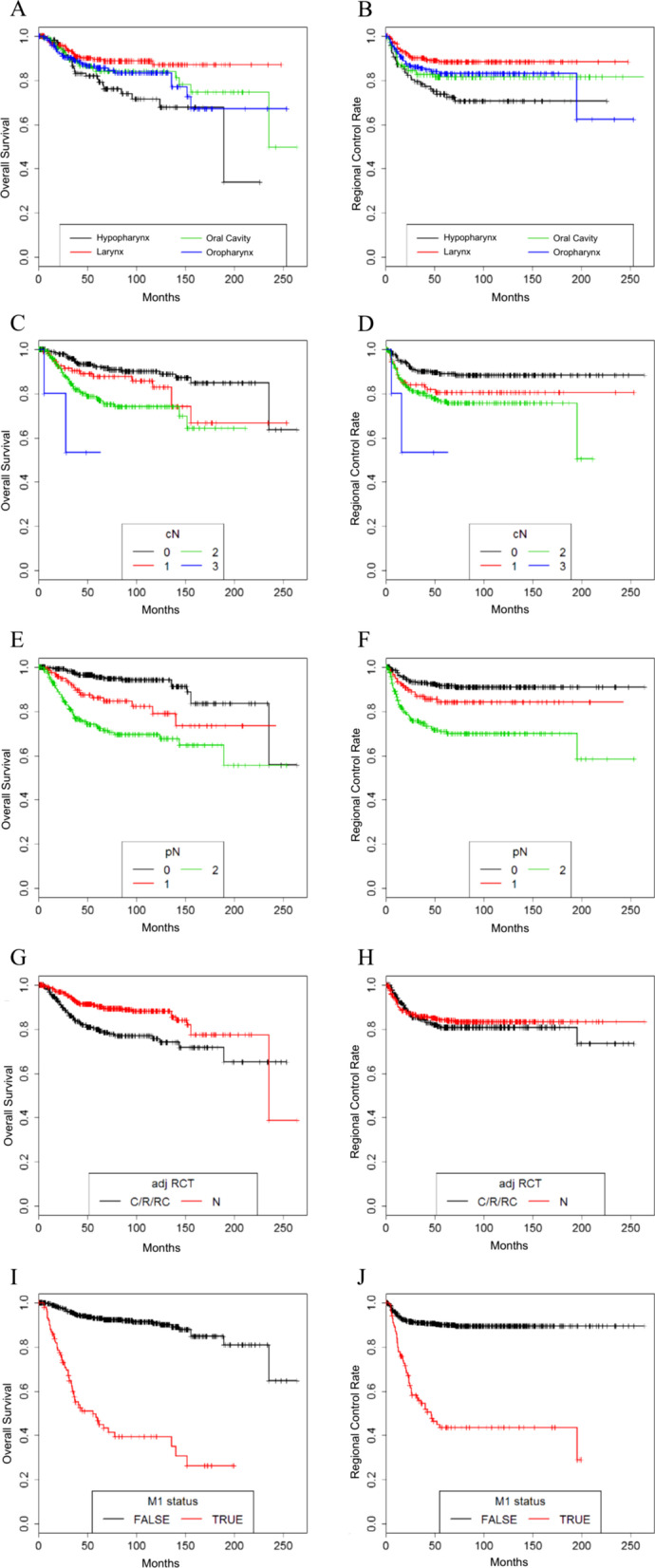
Kaplan Meier Curves: **A** Overall Survival of all patients dependent on tumor site **B** Regional control rate of all patients dependent on tumor site **C** Overall survival of all patients dependent on clinical lymph node category **D** Regional control rate of all patients dependent on clinical lymph node category **E** Overall survival of all patients dependent on pathological lymph node category **F** Regional Control Rate of all patients dependent on pathological lymph node stage **G** Overall survival of all patients dependent on whether they underwent postoperative radio-(chemo-)therapy **H** Regional control rate of all patients dependent on whether they underwent postoperative Radio-(chemo-)therapy **I** Overall survival of all patients dependent on whether they developed distant metastasis **J** Regional control rate of all patients dependent on whether they developed distant metastasis

Out of the 661 patients that were included, 67 patients (10.1%) eventually had lymphatic recurrence of the tumor. 135 (20.4) patients had their primary tumor site in the oral cavity, 179 (27.1%) in the oropharynx, 118 (17.8%) in the hypopharynx and 229 (34.6%) in the larynx. In terms of sides, 158 (23.9%) were situated bilaterally, 23 (3.5%) in the midline, 255 (38.6%) on the right and 225 (34.0%) on the left side. In terms of clinical lymph node category, 261 (39.4%) patients were classified as inapparent/cN0, 111 (16.8%) as N1, 246 (37.2%) as N2 and 5 (0.8%) as N3; in 38 (5.7%) patients, there was no information on clinical lymph node status (cNx). After pathological examination, 302 (45.7%) were classified as pN0, 119 (18.0%) were classified as pN1, and 240 (36.3%) as pN2. 105 (15.6%) patients developed systemic metastasis during follow up, while 558 (84.4%) did not. 291 (44.1%) patients underwent adjuvant radio-(chemo-)therapy after initial surgical tumor therapy, while 370 (55.9%) did not. (Table 1) Respective Kaplan–Meier-diagrams can be found in Fig. 1.

**Table 1:** Basic patient, tumor and surgery characteristics of all patients included in the study by performance of postoperative (chemo-)radiotherapy

Category	Characteristic	No postoperative therapy (n = 370/55.9%)	Postoperative (chemo-)radiotherapy (n = 291/44.1%)	Total (n = 661)
General	Age (years)	57.3 ± 11.0 [15.7–91.6]	56.7 ± 8.8 [37.8–82.3]	57.0 ± 10.1 [15.7–91.6]
	Gender (male/female)	312 (84.3%)/58 (15.7%)	246 (84.5%)/45 (15.5%)	558 (84.4%)/103 (15.6%)
	Follow up (months)	82.0 ± 53.3 [1.1–263.3]	68.0 ± 53.7 [0.3–252.8]	75,8 ± 53.9 [0.3–263.3]
Tumor origin	Oral cavity	78 (21.1%)	57 (19.6%)	135 (20.4%)
	Oropharynx	65 (17.6%)	114 (39.2%)	179 (27.1%)
	Hypopharynx	51 (13.8%)	67 (23.0%)	118 (17.8%)
	Larynx	176 (47.6%)	53 (18.2%)	229 (34.6%)
Tumor side	bilateral	90 (24.3%)	67 (23.0%)	157 (23.8%)
	midline	18 (4.9%)	6 (2.0%)	24 (3.6%)
	right	139 (37.6%)	116 (39.9%)	255 (38.6%)
	left	123 (33.2%)	102 (35.0%)	225 (34.0%)
Tumor category	pT1	60 (16.2%)	26 (8.9%)	86 (13.0%)
	pT2	136 (36.8%)	72 (24.7%)	208 (31.5%)
	pT3	126 (34.1%)	113 (38.8%)	239 (36.2%)
	pT4	48 (13.0%)	80 (27.5%)	128 (19.4%)
clinical Lymph Node Status	cN0	202 (54.6%)	60 (20.6%)	262 (39.6%)
	cN1	62 (16.8%)	49 (16.8%)	111 (16.8%)
	cN2	85 (23.0%)	161 (55.3%)	246 (37.2%)
	cN3	0 (0.0%)	5 (1.7%)	5 (0.8%)
	cNx	21 (5.7%)	16 (5.5%)	37 (5.6%)
pathological Lymph Node Status	pN0	258 (69.7%)	44 (15.1%)	302 (45.7%)
	pN1	59 (15.9%)	60 (20.6%)	119 (18.0%)
	pN2	53 (14.3)	187 (64.3%)	240 (36.3%)
Distant metastasis during follow-up	M0	353 (95.4%)	247 (84.9%)	600 (90.8%)
	M1	17 (4.6%)	44 (15.1%)	61 (9.2%)
Ipsilateral Neck Dissection	any	359 (97.0%)	283 (97.3%)	642 (97.1%)
	Level I	69 (18.6%)	51 (17.2%)	120 (18.2%)
	Level II	356 (96.2%)	257 (88.3%)	613 (92.7%)
	Level III	349 (94.3%)	256 (88.0%)	605 (91.5%)
	Level IV	64 (17.3%)	76 (26.1%)	140 (21.2%)
	Level V	9 (2.4%)	17 (5.8%)	26 (3.9%)
Contralateral Neck dissection	Any	175 (40.6%)	144 (49.5%)	319 (48.3%)
	Level I	34 (9.2%)	20 (6.9%)	54 (8.2%)
	Level II	171 (46.2%)	142 (48.8%)	313 (47.4%)
	Level III	168 (45.4%)	141 (48.5%)	309 (46.7%)
	Level IV	23 (6.2%)	32 (11.0%)	55 (8.3%)
	Level V	3 (0.8%)	4 (1.4%)	7 (1.1%)

Number (share); mean ± standard deviation; [range]

Out of these patients, 644 (97.4%) underwent ispiateral and 319 (48.3%) underwent contralateral neck dissection. On the ipsilateral side, Level I was operated on in 120 (18.2%) patients, Level II in 613 (92.7%), Level III in 605 (91.5%), Level IV in 140 (21.2%) and Level V in 26 (3.9%) patients. On the contralateral side, Level I was operated in 54 (8.2%) patients, Level II in 313 (47.4%), Level III in 398 (46.7%), Level IV in 55 (8.3%) and Level V in 7 (1.1%) patients. 291 of 661 patients received adjuvant (chemo-) radiotherapy as a postoperative treatment (Table 1).

The exact comparisons between the groups in terms of OS and RCR can be found in Table 2. Univariate testing found significant differences in RCR between hypopharynx and larynx as well as hypopharynx and oropharynx, showing lower hazard of regional recurrence for primaries located in the oropharynx (HR 0.6 [0.4–1.0]) and larynx (HR 0.4 [0.2–0.7]). In terms of OS, only the difference between larynx and hypopharynx was significant, demonstrating a better prognosis for laryngeal cancer (HR 0.4 [0.2–0.7]). However, multivariate testing showed a significant influence of the tumor site on both RCR and OS. In terms of pathological tumor category, there was no uni- or multivariate influence on RCR. In terms of OS, pT4a compared to all other categories showed significant differences in univariate testing. Multivariate testing showed a significant influence of pathological tumor category on OS.

**Table 2:** Univariate and multivariate testing of tumor and surgery characteristics and their respective influence on OS and RCR

Stage	Hazard Ratio	p (univariate)	p (multiple)	Hazard Ratio	p (univariate)	p (multiple)
	Regional control rate			Overall survival		
Tumor origin		**< 0.01**	** < 0.01**		**0.02**	**0.01**
Larynx vs. Hypopharynx	0.4 [0.2–0.7]	**< 0.01**		0.4 [0.2–0.7]	** < 0.01**	
Oral Cavity vs. Hypopharynx	0.6 [0.4–1.1]	0.12		0.6 [0.4–1.1]	0.12	
Oropharynx vs. Hypopharynx	0.6 [0.4–1.0]	**0.05**		0.7 [0.4–1.2]	0.16	
Oral Cavity vs. Larynx	1.7 [1.0–3.1]	0.06		1.5 [0.9–2.8]	0.15	
Oropharynx vs. Oral Cavity	0.9 [0.5–1.6]	0.78		1.1 [0.6–1.9]	0.80	
Oropharynx vs. Larynx	1.5 [0.6–1.5]	0.12		1.6 [0.9–2.8]	0.08	
Pathological tumor category		0.13	0.07		** < 0.01**	** < 0.01**
pT2 vs. pT1	1.6 [0.8–3.2]			1.7 [0.8–3.6]	0.16	
pT3 vs. pT1	1.1 [0.5–2.1]			1.2 [0.6–2.7]	0.57	
pT4 vs. pT1	1.7 [0.8–3.4]			3.1 [1.5–6.5]	** < 0.01**	
pT3 vs. pT2	0.6 [0.4–1.0]			0.7 [0.4–1.2]	0.25	
pT4 vs. pT3	0.6 [0.4–1.0]			2.5 [1.5–4.2]	** < 0.01**	
pT4 vs. pT2	1.0 [0.6–1.7]			1.8 [1.1–3.0]	**0.01**	
Clinical lymph node category		**< 0.01**	** < 0.01**		**< 0.01**	** < 0.01**
cN1 vs. cN0	1.9 [1.1–3.4]	**0.03**		1.8 [1.0–3.4]	0.06	
cN2 vs. cN0	2.4 [1.5–3.8]	** < 0.01**		3.0 [1.8–4.9]	**< 0.01**	
cN3 vs. cN0	5.4 [1.3–22.7]	**0.02**		9.4 [2.2–40.0]	**< 0.01**	
cN2 vs. cN1	1.3 [0.7–2.1]	0.40		1.6 [0.9–2.8]	0.09	
cN3 vs. cN1	2.6 [0.6–11.2]	0.20		4.9 [1.1–22.2]	**0.04**	
cN3 vs. cN2	2.3 [0.6–9.6]	0.24		3.0 [0.7–12.3]	0.13	
Pathological lymph node category		** < 0.01**	** < 0.01**		**< 0.01**	**< 0.01**
pN1 vs. pN0	1.9 [1.0–3.5]	**0.05**		2.6 [1.4–5.0]	**< 0.01**	
pN2 vs. pN0	4.0 [2.5–6.4]	** < 0.01**		5.2 [3.1–8.7]	**< 0.01**	
pN2 vs. pN1	2.1 [1.3–3.7]	** < 0.01**		2.0 [1.2–3.3]	**0.01**	
Development of distant metastasis	6.6, [4.5–9.6]	** < 0.01**	0.25	9.8 [6.5–14.6]	**< 0.01**	**< 0.01**
Postoperative (Chemo-) radiation	0.9, [0.6–1.3]	0.51	** < 0.01**	0.5 [0.3–0.8]	**< 0.01**	0.13
Neck dissection ipsilateral	0.7 [0.3–2.0]	0.56	0.37	0.9 [0.3–3.0]	0.93	0.64
Level I ipsilateral	1.3 [0.8–2.0]	0.30	0.25	1.0 [0.6–1.7]	0.96	0.68
Level II ipsilateral	0.5 [0.3–0.9]	**0.01**	0.72	0.5 [0.3–0.9]	**0.02**	0.83
Level III ipsilateral	0.6 [0.3–1.1]	0.07	0.18	0.6 [0.3–1.0]	**0.05**	0.96
Level IV ipsilateral	1.1 [0.7–1.8]	0.62	0.44	1.2 [0.8–1.9]	0.42	0.61
Level V ipsilateral	0.8 [0.2–2.5]	0.68	0.44	0.7 [0.2–2.9]	0.63	0.27
Neck dissection contralateral	1.0 [0.7–1.4]	0.93	0.40	1.3 [0.9–2.0]	0.14	0.79
Level I contralateral	1.6 [0.9–2.9]	0.10	0.17	1.8 [1.0–3.3]	**0.04**	0.07
Level II contralateral	1.0 [0.7–1.4]	0.89	0.88	1.3 [0.9–1.9]	0.23	0.21
Level III contralateral	1.0 [0.7–1.4]	0.96	0.70	1.3 [0.9–1.9]	0.20	0.83
Level IV contralateral	1.2 [0.7–2.4]	0.50	0.48	1.6 [0.8–2.9]	0.16	0.86
Level V contralateral	0.0 [0.0–∞]	0.15	0.23	0.0 [0–∞]	0.20	0.30

Clinical lymph node status showed a significant effect on RCR in any stage compared with cN0 in univariate analysis, demonstrating a higher hazard ratio of regional recurrence in cN + disease. For overall survival this was only the case for cN2 and compared to cN1. Multivariate testing showed a significant influence both for RCR and OS (Table 2).

Pathological lymph node status showed a significant influence on RCR and OS both in univariate and multivariate testing. Development of distant metastasis showed a significant influence on OS both in uni- and multivariate testing and a significant influence on RCR in univariate testing. Adjuvant (chemo-)radiotherapy showed a significant impact on RCR in multivariate and a significant impact on OS in univariate testing. Treated levels during SND showed a significant influence on RCR if ipsilateral Level II was treated in univariate testing. In terms of OS, ipsilateral regions II and III as well as contralateral level I showed a significant influence in univariate testing.

To test whether the observed impact on OS and RCR was dependent on the tumor origin, COX-regression models were fitted that considered the interactions of the tumor origin. These models found a significant influence of surgical treatment of levels I and V ipsilateral and level III contralateral on OS. None of the fitted models revealed a significant influence of any level treated on RCR. (Table 3) Significant findings in these levels indicated that treatment or omission of treatment of these regions may influence overall survival dependent of tumor origin.

**Table 3:** COX-Regression models of operated regions dependent and independent of tumor origin

	Overall survival p (multiple)	Regional control rates p (multiple)
Region	**0.02**	**< 0.01**
Level I ipsilateral	0.93	0.36
Level II ipsilateral	0.08	0.04
Level III ipsilateral	0.69	0.10
Level IV ipsilateral	0.58	0.79
Level V ipsilateral	0.54	0.68
Level I contralateral	**0.02**	0.25
Level II contralateral	0.39	0.80
Level III contralateral	0.65	0.87
Level IV contralateral	0.45	0.50
Level V contralateral	0.20	0.16
**Models with tumor origin interaction**		
Level I ipsilateral	**0.03**	0.81
Level II ipsilateral	0.19	0.07
Level III ipsilateral	0.43	1.00
Level IV ipsilateral	0.47	0.95
Level V ipsilateral	**0.02**	0.16
Level I contralateral	0.66	0.14
Level II contralateral	0.98	0.99
Level III contralateral	**< 0.01**	0.13
Level IV contralateral	0.60	0.68
Level V contralateral	1.00	1.00

Subsequently, we fitted COX-regression models for each tumor region. (Table 4) Treatment of level V ipsilateral or level III contralateral showed no significant influence on OS in any tumor origin. Treatment of level I ipsilateral showed a significant influence on OS when the tumor originated in the oral cavity. The hazard ratio calculated was 0.4 with a confidence interval of 0.2–0.9. This indicated a favorable effect of SND in level I in HNSCC of the oral cavity.

**Table 4:** Influence of neck dissection levels for each tumor region

Parameter	Region			
	Hypopharynx	Larynx	Oral cavity	Oropharynx
Level I ispilateral	4.0 [0.9–17.2] p = 0.06	3.7 [0.5–27.3] p = 0.21	**0.4 [0.2–0.9]** **p = 0.02**	1.9 [0.8–4.7] p = 0.17
Level V ipsilateral	0.0 [0.0–∞] p = 0.16	0.0 [0.0–∞] p = 0.24	0.0 [0.0–∞] p = 0.26	3.0 [0.7–13.1] p = 0.14
Level III contralateral	1.7 [0.7–3.7] p = 0.22	1.2 [0.5–2.8] p = 0.68	1.7 [0.7–4.0] p = 0.21	1.4 [0.7–2.9] p = 0.38

## Discussion

This retrospective analysis of data basing on prospective documentation of consecutive cases of head and neck cancer identified factors for regional failure and general outcome. Firstly, we found that the tumor site has both a significant effect on regional control rates as well as overall survival. Moreover, both clinical and pathological lymph node status shows a significant impact on both overall survival and regional control rates. Tumor size, as indicated by the pT-categories, showed a significant impact on OS but not RCR. Finally, the value of ipsilateral dissection of level I in oral cavity cancer was emphasized.

A strength of this work is a high number of 661 cases and a considerably long follow-up-time of 78.9 ± 106.4 months. Other recent studies report 84 to 123 patients [13–15] with 12 to 30 months of follow-up [13, 15]. However, since only a core dataset was documented prospectively, this study is subject to various kinds of bias typical for retrospective analyses, including systematic lack of data. Most importantly, HPV-status was only assessed systematically in recent years at the study center and could not be included in the present analysis. Clinical decisions, for example regarding the inclusion or omission of a particular neck level into a neck dissection might have been due to factors that cannot be deducted in retrospect from the available data. Additionally, during the long period of data collection from 1986 to 2017 shifting practices for indication or performance of neck dissection and postoperative treatment may have taken place even if this shift cannot be clearly determined from the available documentation.

Another limitation of our study is the inclusion of head and neck tumors of various stages and all subsites of the upper aerodigestive tract. Consecutively, this population exhibits a considerable heterogeneity. Reason for that was the assumption, that the basic principles of metastasizing apply to the head and neck cancers in general and therefore a more global view as it was chosen here might yield results of interest. It limits, however, the conclusions that can be drawn from our results and necessitates prospective investigations on more heterogeneous subpopulations in the future to affirm the findings presented here.

It has been established that SND is the appropriate treatment for regional spread in HNSCC. D’Cruz and colleagues found in a randomized, prospective study that elective SND in early HNSCC of the oral cavity is superior to a wait-and see approach and subsequent SND in the case of positive lymph nodes [16]. An influence of tumor site on regional control and overall survival is mainly due to the fact that laryngeal and oropharyngeal HNSCC are showing a significant impact on OS and RCR. This is not surprising as hypopharyngeal carcinomas have a very poor overall prognosis; commonly they are diagnosed in a late stage and with advanced nodal stage [17]. Moreover, they often show a tendency for more dedifferentiated carcinomas and occult cervical metastasis [18]. These factors make surgical management of hypopharyngeal carcinoma significantly more demanding, with a distinct possibility of occult tumor cells remaining on site, impairing both RCR as well as OS. A heavy influence of clinical and pathological lymph node status is not surprising, given that the greater the load of tumor cells in the neck, the greater the probability of cells not being reached by surgery and eventually creating the foundation for recurrence of cancer. Subsequently, this observation is very much in line with literature addressing this topic: Layland and colleagues found in over 3.800 patients that advanced neck stages are associated with poorer OS in all tumor sites [18]. Ambrosch and colleague found in a very similar collective to that at hand that advanced nodal stage as well as extracapsular extension are associated with a greater probability for regional recurrences [19]. While the impact of tumor size on OS is not surprising and has been evaluated in large-scale studies, [20] the missing correlation between tumor size and RCR in our sample is somewhat surprising. We believe that this observation may be explained by the (relatively) effective removal of the primary tumor by TLM [21–23].

When considering the individual neck dissection levels that were included during surgery, we found in the models that included an interaction with the tumor site that levels I and V ipsilaterally as well as level III contralaterally showed an influence on overall survival upon closer examination. When correcting those models for the tumor site, we only found a significant impact on overall survival on ipsilateral neck dissection in contralateral level I in HNSCC of the oral cavity. Fittingly, the respective level has been included in the prospective study by D’Cruz and colleagues that found elective neck dissection to be superior to a wait-and-see approach [16]. The recommendation for elective and therapeutic surgical treatment of level I in oral cavity SCC has already been given in a comprehensive clinical practice guideline [24]. While those models that did correct for tumor origin did not indicate a statistically significant influence on OS, it is our conviction that inclusion of these levels in primary SND may still be beneficiary and should be considered by surgeons. Fittingly, Frohwitter and colleagues found that affection of ipsilateral levels IV and V considerably decreases overall survival in HNSCC of the oral cavity [14].

Despite the significant impact of levels I and V ipsilaterally as well as level III contralaterally on overall survival in the total patient cohort, a significant difference only persisted for oral cavity tumors when broken down by site of primary tumor. This is surprising at first sight, since many laryngeal tumors show a clear benefit by elective neck dissection regarding regional control. However, that is mainly true for supraglottic and transglottic tumors due to their comparably high rate of occult metastases [25]. We assume that a potentially relevant finding regarding neck dissection levels for laryngeal tumors could not be demonstrated due to the heterogeneity of our dataset. Furthermore, subsites like supraglottic, glottic and transglottic larynx should most likely be analyzed separately.

Also most likely due to heterogeneity, the definite relevance of level V dissection may be impossible to assess in our study population. A recent review puts the occurrence of occult metastases in level V in a neck that is affected by positive nodes at other levels at 2.56% [26]. It seems likely that this threshold is too low for the detection of a significant difference in any subgroup reported here. Specifically, a more differentiated consideration of cN0 versus cN + status may be necessary than it was possible here.

## Conclusion

In conclusion, our results support the longstanding view that SND is effective both in preventing nodal relapse in cases without evidence for regional spread as well as treating manifest cervical metastases. The study at hand is—to the best of the authors knowledge—the largest study to comprehensively analyze the treated levels in SND dependent on tumor size and origin. The dissection of ipsilateral level I should be a special focus in patients with HNSCC of the oral cavity. The definite value of this part of selective neck dissection in various clinical situations has to be established in prospective evaluations of more homogenous patient cohorts.

## Dataset

The authors of this manuscript declare that the data at hand have not been published, submitted or used in any other manuscript elsewhere.

## Data Availability

The dataset generated and analysed during the current study is not publicly available, since the local ethics committee explicitly discouraged the publication of individual data in its statement due to data privacy. The corresponding author will provide an anonymized dataset on reasonable request.

## Abbreviations

HNSCC: Head and neck squamous cell carcinoma
OS: Overall survival
RCR: Regional control rate
RND: Radical neck dissection
SND: Selective neck dissection
TLM: Transoral laser microsurgery
